# Supplementary material for: Stacking Model‐Based Classifiers for Dealing With Multiple Sets of Noisy Labels
Source: Biom J. 2025 Mar 12;67(2):e70042. doi: 10.1002/bimj.70042 (PMC11898607; doi:10.1002/bimj.70042)
Supplement: Supplementary file 1 — Supporting Information [file BIMJ-67-e70042-s001.zip › density-based-ensemble-model-main/competitors_real_data/netlab-master/netlab-master/help/rbfbkp.htm]

Netlab Reference Manual rbfbkp


# rbfbkp

## Purpose

Backpropagate gradient of error function for RBF network.

## Synopsis

```
g = rbfbkp(net, x, z, n2, deltas)
```

## Description

`g = rbfbkp(net, x, z, n2, deltas)` takes a network data structure
`net` together with a matrix `x` of input vectors, a matrix
`z` of hidden unit activations, a matrix `n2` of the squared
distances between centres and inputs, and a matrix `deltas` of the
gradient of the error function with respect to the values of the
output units (i.e. the summed inputs to the output units, before the
activation function is applied). The return value is the gradient
`g` of the error function with respect to the network
weights. Each row of `x` corresponds to one input vector.

This function is provided so that the common backpropagation algorithm
can be used by RBF network models to compute
gradients for the output values (in `rbfderiv`) as well as standard error
functions.

## See Also

`rbf`, `rbfgrad`, `rbfderiv`

---

**Pages:**
Index


---

Copyright (c) Ian T Nabney (1996-9)
